# Mitochondrial reactive oxygen species regulate HIF-1α stabilization and methylglyoxal accumulation in classically activated mouse macrophages

**DOI:** 10.1097/IN9.0000000000000085

**Published:** 2026-08-04

**Authors:** Daniel Prantner, Mark A. Watson, Martin D. Brand, Jules C. Paton, Stefanie N. Vogel

**Affiliations:** 1 Department of Microbiology and Immunology, University of Maryland School of Medicine, Baltimore, MD, USA; 2 Buck Institute for Research on Aging, Novato, CA, USA

**Keywords:** electron transport, glycation, hypoxia-inducible factor, inflammation, innate immunity macrophage, mitochondrial respiratory chain complex, nitric oxide synthase

## Abstract

**Background:**

The economic and medical burden of sepsis worldwide underscores the need for novel therapeutics. Early sepsis involves dramatic metabolic changes. Classically activated macrophages, stimulated with lipopolysaccharide and interferon-γ, shift their metabolism to glycolysis. The reactive glycolytic metabolite, methylglyoxal, accumulates and has been associated with adverse outcomes in sepsis. We previously demonstrated that hypoxia-inducing factor-1α (HIF-1α) contributes to methylglyoxal accumulation. Treatment with lipopolysaccharide or interferon-γ individually stabilized HIF-1α protein; however, co-stimulation with both lipopolysaccharide and interferon-γ accelerated HIF-1α stabilization, implying a shared upstream mediator. Therefore, we sought to characterize mechanisms underlying methylglyoxal accumulation.

**Methods:**

Quantitative polymerase chain reaction and immunoblotting were used to analyze HIF-1α expression in classically activated primary mouse macrophages.

**Results:**

*Nos2* expression was induced by lipopolysaccharide or interferon-γ and markedly enhanced by combined treatment, possibly linking inducible nitric oxide synthase (iNOS) activity to HIF-1α stabilization. Inhibiting iNOS with l-NG-Nitro arginine methyl ester (l-NAME) reduced HIF-1α stabilization in a dose-dependent manner. Mitochondrial reactive oxygen species (mROS), generated following nitric oxide inhibition of cytochrome oxidase, similarly contributed to HIF-1α stabilization, as shown by the effects of suppressors of ROS production by mitochondrial complex I (S1QEL1.1) and III (S3QEL1.2) and the compartment-specific antioxidant Mito-TEMPO. Blocking mROS also decreased *Il1b, Il6, and Cxcl10* expression in activated macrophages, supporting a broader impact on inflammation. Additionally, S1QEL1.1 treatment reduced accumulation of methylglyoxal.

**Conclusions:**

These data support a model in which nitric oxide-mediated mitochondrial dysfunction increases mROS, promoting HIF-1α stabilization and methylglyoxal accumulation, thereby shaping macrophage inflammatory responses. Thus, targeting mROS may offer a therapeutic strategy to improve sepsis outcomes.

## 1. Introduction

Sepsis is defined medically as life-threatening organ dysfunction caused by a dysregulated host response to infection ^[1]^. Approximately 166 million cases of sepsis occur annually worldwide, with over 21 million all-cause sepsis deaths ^[2]^. Unfortunately, effective treatment options remain scarce. Multiple immune cells, including macrophages, contribute to the development of host pathology ^[3]^. Early stages of sepsis are accompanied by dramatic changes in host metabolism and inflammation, suggesting that these processes may be linked. Due to the plasticity of macrophages, their phenotype can be extensively regulated depending on their polarization state ^[4]^. In classically activated mouse macrophages generated by stimulation with lipopolysaccharide (LPS) and interferon (IFN)-γ, a metabolic shift from homeostatic conditions is observed, resulting in impaired oxidative phosphorylation and the exclusive use of glycolysis (Warburg effect) to satisfy the energy requirements of the cell ^[5]^. This shift towards glycolysis is accompanied by the accumulation of the highly reactive metabolite, methylglyoxal, in these macrophages, ^[5]^ which is potentially a byproduct of increased triose phosphate isomerase activity ^[6]^. Methylglyoxal can cause glycation-induced protein dysfunction, and increased levels of methylglyoxal have been proposed as a biomarker for adverse outcomes in sepsis ^[7]^. Additionally, antagonism of methylglyoxal in experimental mouse models of sepsis has been shown to be protective, potentially by regulating capillary leakage ^[8]^. Blocking glycolysis with the glucose analog 2-deoxyglucose limits the induction of proinflammatory cytokines in response to LPS ^[9,10]^ and in LPS + IFN-γ-treated, classically activated macrophages ^[11]^. Overall, these findings indicate that targeting the metabolic shift toward increased glycolysis in macrophages may be beneficial during sepsis or other inflammatory conditions.

Previous studies have indicated that the proinflammatory state of macrophages is mediated by the transition of mitochondria from adenosine triphosphate (ATP) generation to reactive oxygen species (ROS) production ^[12]^. Specifically, preventing mitochondrial ROS (mROS) production through pharmacological inhibition of succinate oxidation was shown to mitigate LPS-induced lethality in mice ^[12]^, suggesting that inhibition of mitochondrial metabolism may have therapeutic benefit. However, a causal role of fatty acid oxidation, which can drive mitochondrial respiration through production of acetyl-CoA, on macrophage activation has been questioned based on discrepant results obtained by pharmacologic vs genetic methods of impairing fatty acid oxidation ^[13]^. This also suggests that previous data derived using pharmacologic means should be confirmed using the most specific reagents available.

Hypoxia-inducing factor-1α (HIF-1α) is a master transcriptional regulator of glycolytic pathways and serves to mitigate the effects of low oxygen tension and the resulting decreased ATP generation caused by impaired oxidative phosphoryla-tion ^[14,15]^. It is also an important regulator of macrophage function ^[16]^, including macrophage polarization ^[17]^. The half-life of HIF-1α under conditions of normal physiologic oxygen is ~5 minutes ^[18]^. Rapid cellular turnover of HIF-1α is mediated by hydroxylation of a conserved proline residue by a family of enzymes known as prolyl hydroxylases (PHDs) ^[19]^, which leads to proteasomal degradation of HIF-1α ^[20]^. Hypoxia induces stabilization of HIF-1α due to the inability of the PHD proteins to form hydroxyl bonds in the absence of molecular oxygen, resulting in changes in gene expression such as the upregulation of the glucose transporter *Glut1* ^[21,22]^. Although anaerobic conditions are the canonical cause of HIF-1α stabilization, toll-like receptor (TLR) 4-dependent signaling or stimulation with other growth factors can stabilize HIF-1α even under aerobic conditions ^[23,24]^. Due to its essential role in the response to LPS in a mouse model ^[25]^, HIF-1α has been extensively studied in the context of Gram-negative sepsis ^[26]^.

We previously demonstrated that methylglyoxal accumulation was mediated by HIF-1α in primary mouse macrophages when stimulated with LPS alone or in conjunction with IFN-γ ^[11]^. However, the mechanism underlying the stabilization of HIF-1α under aerobic conditions remained unclear. Therefore, we sought to characterize how HIF-1α is stabilized in classically activated macrophages, including the potential contributions of reactive species and the mitochondria, to identify targets to reduce deleterious methylglyoxal accumulation.

## 2. Methods

### 2.1 Reagents and antibodies

*Escherichia coli* K235 LPS was prepared as previously described ^[27]^. Recombinant mouse IFN-γ (# 485-MI-100/CF) was purchased from R&D Systems (Minneapolis, MN, USA). Antibodies specific for HIF-1α (clone #D1S7W) and β-actin (#4967) were purchased from Cell Signaling (Danvers, MA, USA). Antibodies specific for the methylglyoxal-derived hydroimidazolones MG-H1 and MG-H3 were kindly provided by Dr. David Spiegel (Yale University, CT, USA) ^[28]^. The small compounds l-NG-Nitro arginine methyl ester (l-NAME) (#80210) and Mito-TEMPO (#16621) were purchased from Cayman Chemical (Ann Arbor, MI, USA). Nitrite, a stable breakdown product of nitric oxide (NO), was measured using the Griess assay ^[29]^. Sodium nitrite was purchased from Sigma-Aldrich (St. Louis, MO, USA) and used as the standard for this assay. S1QEL1.1 and S3QEL1.2 were provided by Dr. Martin D. Brand (Buck Institute, CA, USA) ^[30,31]^.

### 2.2 Mice and macrophage isolation

All experiments performed on mice were approved by the Institutional Animal Care and Use Committee at the University of Maryland, Baltimore, for animal use protocols 0622010 (July 20, 2022) and 00003511 (August 13, 2025). Thioglycollate-elicited mouse peritoneal macrophages were obtained by peritoneal lavage 4 days after intraperitoneal injection with 2 mL of sterile thioglycollate (Remel, Lenexa, KS, USA) as previously described ^[32]^. Mouse macrophages were purified and cultured as previously described ^[33]^ and were stimulated with LPS (10 ng/mL) alone, IFN-γ (20 ng/mL) alone, or LPS plus mouse IFN-γ to polarize the cells to a classically activated (M1) phenotype ^[5]^.

### 2.3 Analysis of protein expression by Western blotting

Cell lysates obtained from macrophages in tissue culture were extracted with cell lysis buffer (Cell Signaling Technology, Danvers, MA, USA). Before immunoblotting, all lysates were centrifuged at 12,000 RPM for 10 minutes at 4 °C to pellet insoluble cell debris. The resulting lysates were separated by sodium dodecyl sulfate-polyacrylamide gel electrophoresis (SDS-PAGE) immediately or frozen as single-use aliquots at −80 °C. Before separation by SDS-PAGE, cell lysates were mixed with an equal volume of 2× Laemmli sample buffer (Bio-Rad, Hercules, CA, USA) and the mixtures heated to 95 °C for 5 minutes. Continuous 10% or 12% polyacrylamide pre-cast gels (Bio-Rad, Hercules, CA, USA) were used for the separation of the individual samples by SDS-PAGE. Immediately after separation, proteins were transferred from the gel to a polyvinyl difluoride (PVDF) membrane in transfer buffer consisting of TRIS-Glycine buffer (Bio-Rad, Hercules, CA, USA) and 20% methanol at 4 °C, using a constant voltage of 100 volts for 1 hour. The PVDF membrane was dried to allow complete adherence of the proteins to the membrane. Before probing the membrane with specific antibodies directed against HIF-1α and β-actin, the membrane was washed with Tris-buffered saline (TBS) and blocked with TBS containing 0.1% (v/v) Tween 20 and 5% (w/v) blotting grade blocker (#1706404, Bio-Rad, Hercules, CA, USA). To quantify signal intensities on blots, densitometry was performed as previously described ^[34]^. Briefly, an open-source software program, ImageJ ^[35]^, was used for densitometry by calculating the pixel intensity across the length of each lane of a western blot, creating a graph of intensity vs position. Calculating the area under the curve of these graphs with the software program provides a surrogate measure of the total protein present in an individual band once the signal corresponding to an empty lane is subtracted out to compensate for background intensity. Finally, all intensities were normalized to the housekeeping protein, β-actin, in the same lane using the same ImageJ analysis as described above to account for variations in protein loading. Each blot chosen for publication is representative of three independent experiments with similar outcomes. All blots will be made publicly available after publication.

### 2.4 Quantitative reverse transcription polymerase chain reaction

Synthesis of cDNA from RNA for use in quantitative reverse transcription polymerase chain reaction (qRT-PCR) has been previously described ^[33]^. Gene expression was analyzed using QuantStudio 3 (Applied Biosystems, Carlsbad, CA, USA) along with the 2× Power SYBR green mix PCR master mix (Life Technologies Corporation, Carlsbad, CA, USA). All the individual cytokine gene-specific primers used to detect differences in gene expression have previously been published ^[33]^. The total amount of mRNA was calculated using the Comparative ∆∆Ct method with *Hprt* as the housekeeping gene ^[36]^, allowing the expression of data as fold-induction compared with levels of mRNA detected in control cells.

### 2.5 Statistical analysis

Experiments that yielded gene expression data as the primary readout were performed using duplicates per sample for each group within each experiment. The average value obtained from the duplicate samples was calculated. Specifically, three independent experiments were performed, and the means of these duplicate values for each of the three independent experiments were collected. These collected mean values were graphed on the same axis, with each mark representing an independent experiment. The number of independent experiments is also indicated in each figure legend. For gene expression analyses of experiments, values from the multiple experiments were analyzed with a one-way analysis of variance (ANOVA) followed by a Tukey’s post- hoc test using GraphPad Prism 10 (GraphPad Software Inc, La Jolla, CA, USA) as previously described ^[5]^. For immunoblot analysis, densitometric values were calculated using ImageJ. Similar to our analysis of RNA expression data, densitometry results from individual immunoblot experiments were repeated and compiled as previously described ^[11]^. One-way ANOVAs were used to test for significance between experimental groups within the data set. The individual values were graphed together on the same axis to illustrate statistical differences, while a representative blot was displayed adjacent to the graph. A *P* value < 0.05 was used as the threshold to determine statistical significance for all analyses.

### 2.6 Ethical approval

All experiments performed on mice were approved by the Institutional Animal Care and Use Committee at the University of Maryland, Baltimore, for animal use protocols 0622010 (July 20, 2022) and 00003511 (August 13, 2025). All animal experiments were conducted and reported in compliance with the ARRIVE 2.0 guidelines.

## 3. Results

### 3.1 Inhibition of NOS enzymes inhibits HIF-1α stabilization in classically activated macrophages

**Figure 1.**
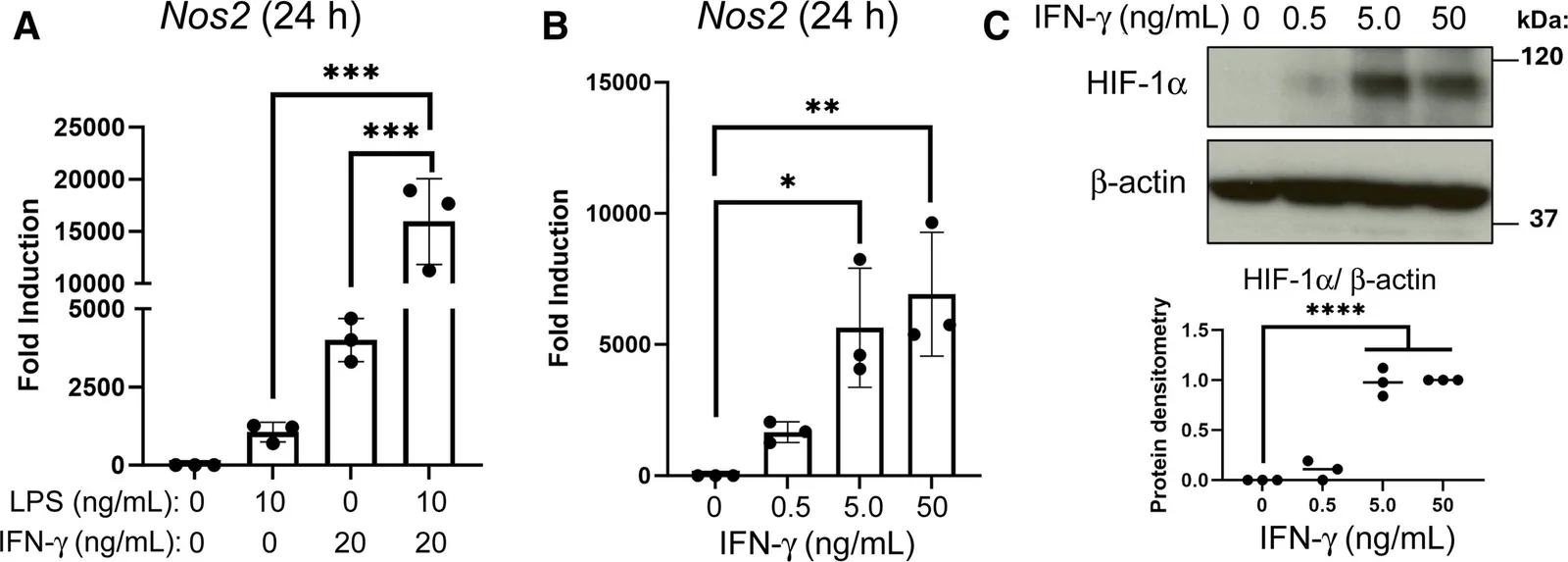
**Expression of *Nos2* correlates with aerobic stabilization of HIF-1α**. (A) Mouse peritoneal macrophages were stimulated with either LPS (10 ng/mL) or IFN-γ (20 ng/mL), individually or in combination, for 24 hours. Expression of *Nos2* was quantified by qRT-PCR. (B) Mouse peritoneal macrophages were stimulated with increasing doses of IFN-γ for 24 hours. Expression of *Nos2* was quantified by qRT-PCR. (C) In parallel with (B), whole cell lysates were isolated at 24 hours poststimulation, and these lysates were subjected to western blot analysis, probed with antibodies specific for HIF-1α and β-actin. Data in panels (A) and (B) represent the compilation of three independent experiments, with each dot representing the “fold induction” compared to untreated cells calculated from each of these experiments. Error bars portray the standard error of the means (SEM). * denotes *P* < 0.05. ** denotes *P* < 0.01. *** denotes *P* < 0.001. The images in (C) portray representative blots from three independent western blot experiments. The chart underneath the blots portrays the mean densitometric results from each of these three experiments. **** denotes *P* < 0.0001. HIF-1α, hypoxia-inducing factor-1α; NOS, nitric oxide synthase.

**Figure 2.**
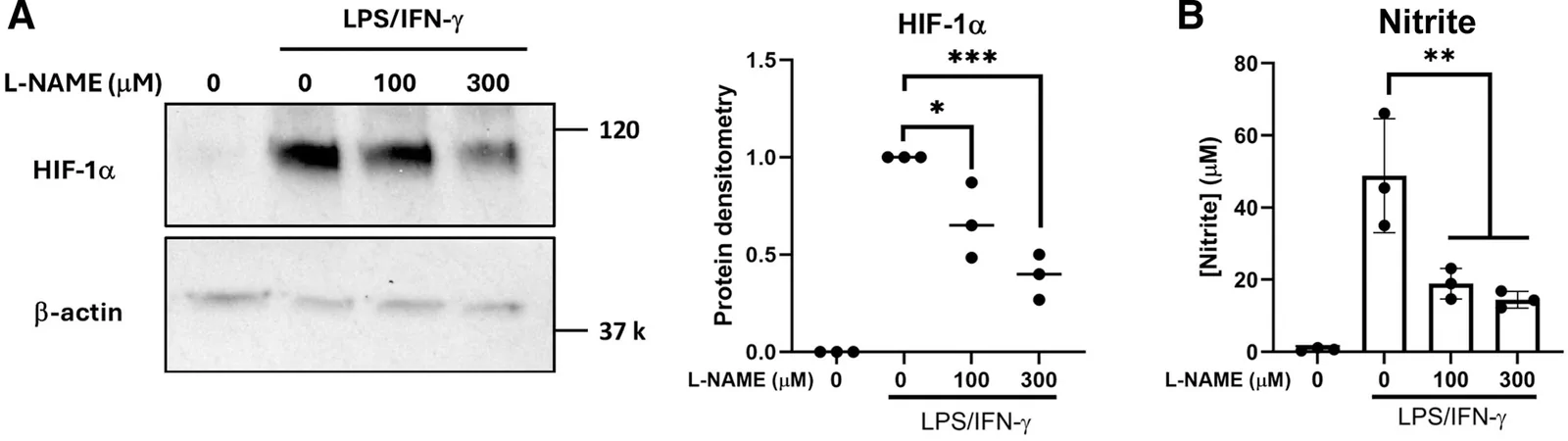
**Inhibition of NOS enzymes represses aerobic stabilization of HIF-1α in classically activated macrophages.** Mouse peritoneal macrophages were pretreated for 30 minutes with 0, 100, or 300 μM l-NAME. Macrophages were then stimulated with both LPS (10 ng/mL) and IFN-γ (20 ng/mL) as indicated by the black bar for 24 hours. (A) Whole cell lysates were isolated at 24 hours poststimulation and were analyzed by western blotting, probed with antibodies specific for HIF-1α and β-actin. The immunoblot images portray representative blots from three independent experiments. The graph below the blots portrays the mean densitometric results from each of these three experiments. * denotes *P* < 0.05. *** denotes *P* < 0.001. (B) In parallel to (A), nitrite levels in the macrophage culture supernatants were determined using the Griess assay. ** denotes *P* < 0.01. HIF-1α, hypoxia-inducing factor-1α; l-NAME, l-NG-Nitro arginine methyl ester; NOS, nitric oxide synthase.

We previously demonstrated that LPS or IFN-γ are individually capable of stabilizing HIF-1α in murine macrophages, and co-treatment of macrophages with LPS and IFN-γ resulted in more rapid and synergistic HIF-1α stabilization ^[11]^. Previous studies have indicated that increased inducible nitric oxide synthase (iNOS), a product of the *Nos2* gene, leads to the production of NO, which, in turn, modulates oxidative phosphorylation ^[37]^. Additionally, classically activated macrophages that were conditionally deficient for HIF-1α exhibited significantly decreased expression of *Nos2* ^[11]^. *Nos2* gene expression was induced by treatment with LPS or IFN-γ individually, and dual treatment had a synergistic effect (Figure 1A), consistent with prior results examining NO production in a mouse macrophage cell line ^[38]^. In macrophages stimulated exclusively with IFN-γ, *Nos2* expression correlated with HIF-1α stabilization (Figure 1B,C). Despite this correlation, a causative relationship between *Nos2* induction and HIF-1α stabilization remains unproven. Therefore, classically activated macrophages were treated with the nonselective NOS inhibitor, l-NAME, which has been previously shown to decrease NO production ^[39,40]^. l-NAME treatment dose-dependently reduced HIF-1α stabilization induced by LPS in combination with IFN-γ (Figure 2A). This decrease in HIF-1α stabilization was associated with a significant decrease in NO production (Figure 2B), indicating a potential relationship between NOS induction and HIF-1α stabilization.

### 3.2 Targeting ROS generation by the mitochondria inhibits HIF-1α stabilization in classically activated macrophages

**Figure 3.**
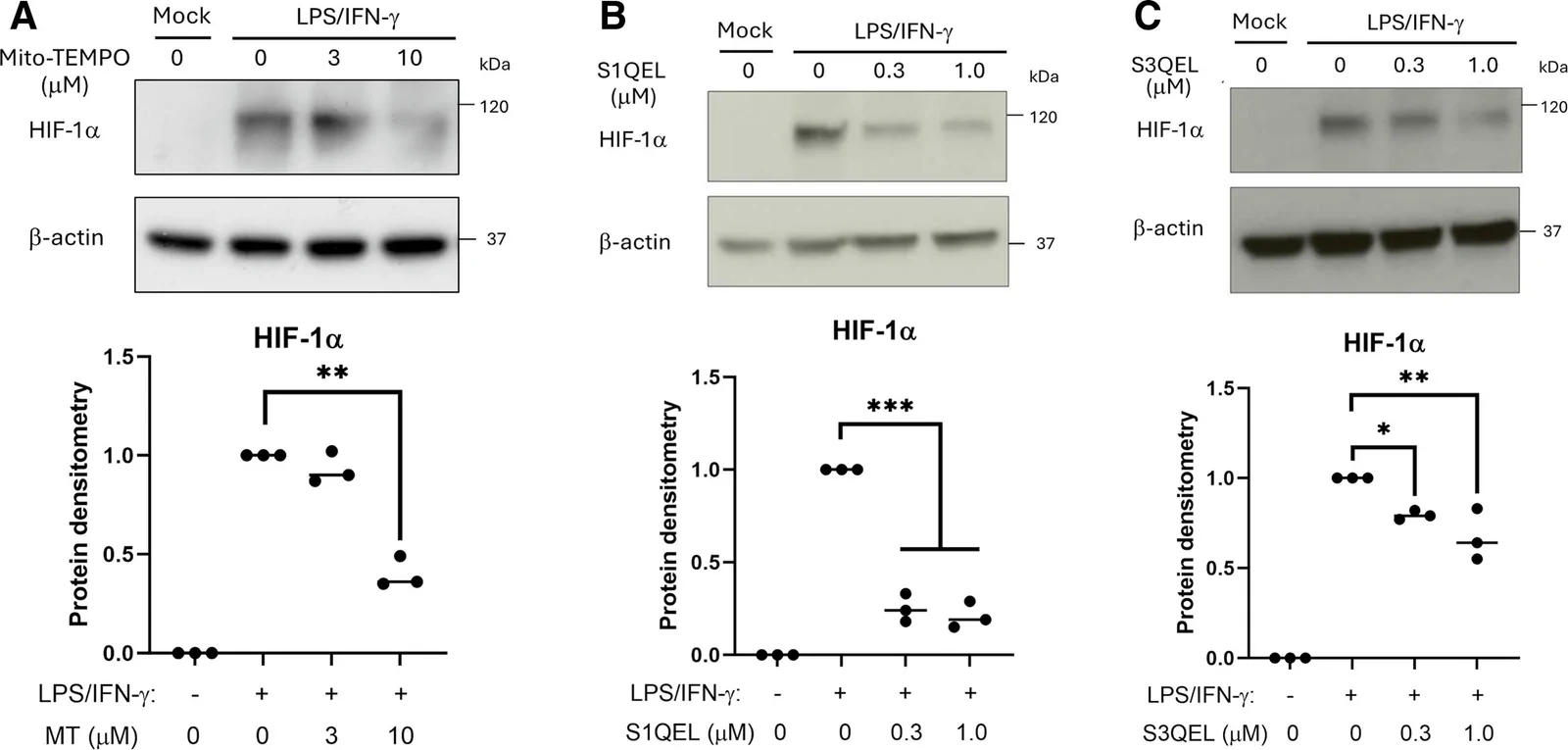
**Inhibition of mROS represses aerobic stabilization of HIF-1α in classically activated macrophages.** Mouse peritoneal macrophages were stimulated for 24 hours with LPS (10 ng/mL) and IFN-γ (20 ng/mL) as indicated by the black bar. Thirty minutes before these treatments, cells were treated with the indicated doses of (A) Mito-TEMPO, (B) S1QEL1.1, or (C) S3QEL1.2. Whole cell lysates were isolated at 24 hours poststimulation and these lysates were subjected to western blotting, probed with antibodies specific for HIF-1α and β-actin. The immunoblot images for all panels portray representative blots from three independent experiments. The graphs underneath the blots portray the mean densitometric results from each of these three experiments. ** denotes *P* < 0.01. *** denotes *P* < 0.001. HIF-1α, hypoxia-inducing factor-1α; mROS, mitochondrial reactive oxygen species.

In cell culture, targeting mitochondrial complex IV activity with NO has been suggested to lead to generation of mROS, perhaps by inducing reverse electron flow through the mitochondrial electron transport chain ^[41]^. However, it is more likely that NO causes strong reduction of the electron transport chain in the presence of high proton motive force generated by hydrolysis of glycolytic ATP. Overall, this finding suggests that mROS generation is one possible pathway downstream of NO that may contribute to HIF-1α stabilization. To address this possibility, classically activated macrophages were initially treated with a mitochondrial-targeted antioxidant, Mito-TEMPO. As has been demonstrated previously with LPS-treated bone marrow-derived macrophages ^[12]^, addition of Mito-TEMPO dose-dependently decreased HIF-1α stabilization in classically activated macrophages (Figure 3A). However, the possibility exists that this may be due to an off-target effect or due to a generalizable effect on cellular ROS pools. Therefore, ROS was targeted more specifically with reagents that suppress mROS production from either complex I (S1QEL1.1) or complex III (S3QEL1.2) of the electron transport chain ^[30,31]^. Both S1QEL1.1 and S3QEL1.2 significantly decreased HIF-1α stabilization in classically activated macrophages (Figure 3B,C). While both S1QEL1.1 and S3QEL1.2 suppress HIF-1α stabilization, there is \a greater reduction in HIF-1α stabilization with the use of S1QEL1.1, indicating that complex I is possibly the main source of ROS driving HIF-1α stabilization. Alternatively, the differential inhibitory strengths of these two inhibitors may underlie this effect.

### 3.3 Treatment of macrophages with mROS inhibitors regulates gene expression in classically activated macrophages

**Figure 4.**
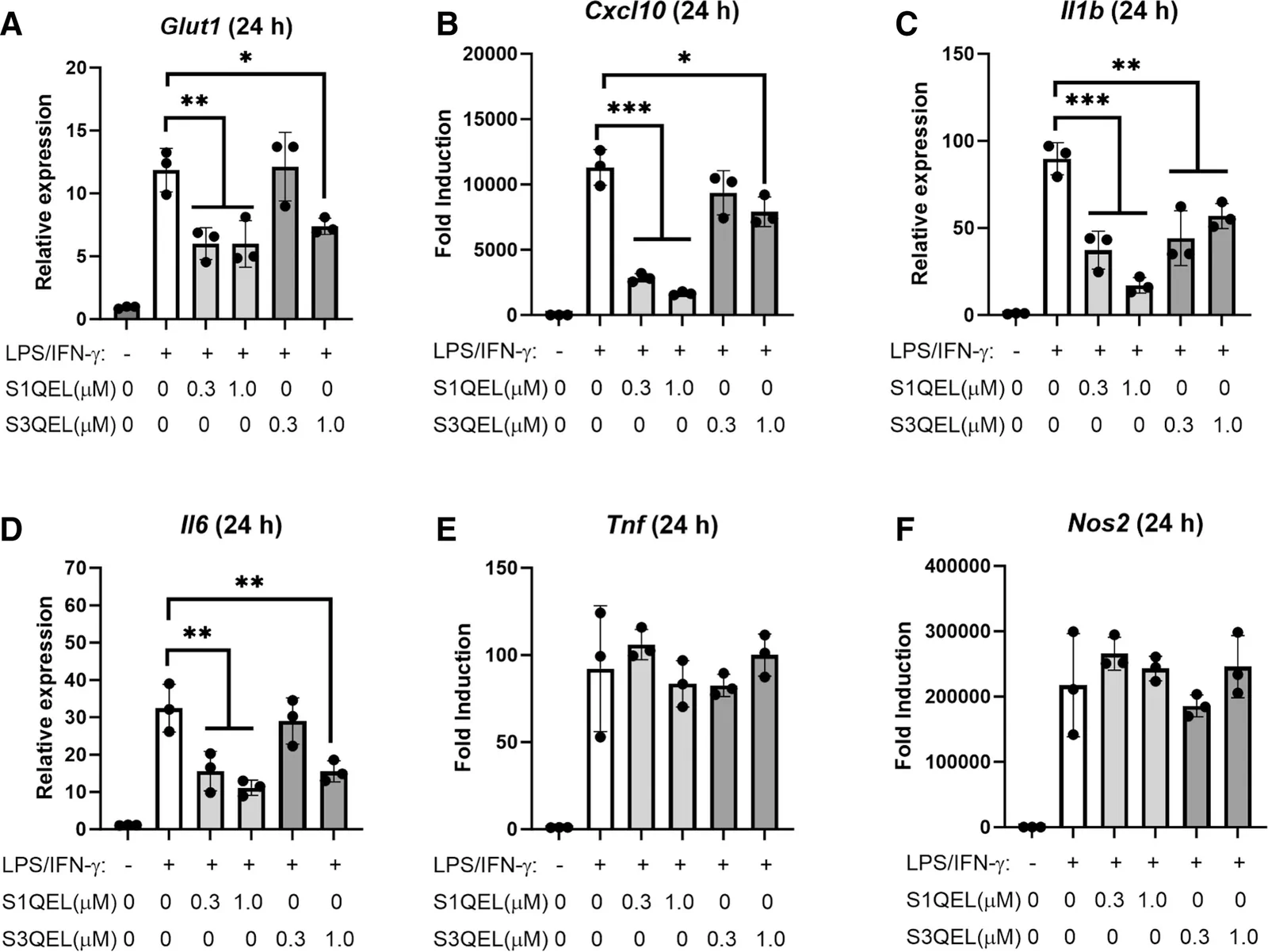
**Inhibition of mROS decreases expression of a subset of cytokines associated with inflammation in classically activated macrophages.** WT mouse peritoneal macrophages were left untreated or stimulated simultaneously with LPS (10 ng/mL) and IFN-γ (20 ng/mL) for 24 hours, where indicated. Thirty minutes before this treatment, cells were treated with either vehicle control (DMSO) or the indicated doses of S1QEL1.1 or S3QEL1.2. Expression of *Glut1* (A), *Cxcl10* (B), *Il1b* (C), *Il6* (D), *Tnf* (E), and *Nos2* (F) mRNAs were quantified by qRT-PCR. Data in (A–F) represent the compilation of the “fold induction” values compared with untreated cells from three independent experiments, with each symbol representing the mean value +/− SEM calculated from these individual experiments. * denotes *P* < 0.05. ** denotes *P* < 0.01. *** denotes *P* < 0.001. mROS, mitochondrial reactive oxygen species.

Targeting host metabolism has been speculated to be beneficial in situations where deleterious inflammation is causing host pathology ^[42]^. Therefore, we examined the effects of inhibitors that target mROS on alteration of inflammatory gene expression in classically activated macrophages. S1QEL1.1 and S3QEL1.2 suppress ROS production by mitochondrial complexes I and III, respectively ^[30,31]^. In macrophages stimulated with LPS and IFN-γ, S1QEL1.1 and S3QEL1.2 treatment significantly decreased expression of the target gene *Glut1* (Figure 4A). However, S1QEL1.1 elicited stronger and more potent inhibition than S3QEL1.2 of gene expression of the chemokine gene, *Cxcl10* (Figure 4B), which we had previously identified as being strongly HIF-1α-dependent in classically activated macrophages ^[11]^. Similarly, S1QEL1.1 and S3QEL1.2 also significantly decreased expression of *Il1b* and *Il6*, but not *Tnf* (Figure 4C–E). S3QEL-mediated inhibition was observed only at the highest concentration used. Neither S1QEL1.1 nor S3QEL1.2 decreased *Nos2* expression in classically activated macrophages (Figure 4F), suggesting that these compounds do not exert their effect through regulation of NO.

**Figure 5.**
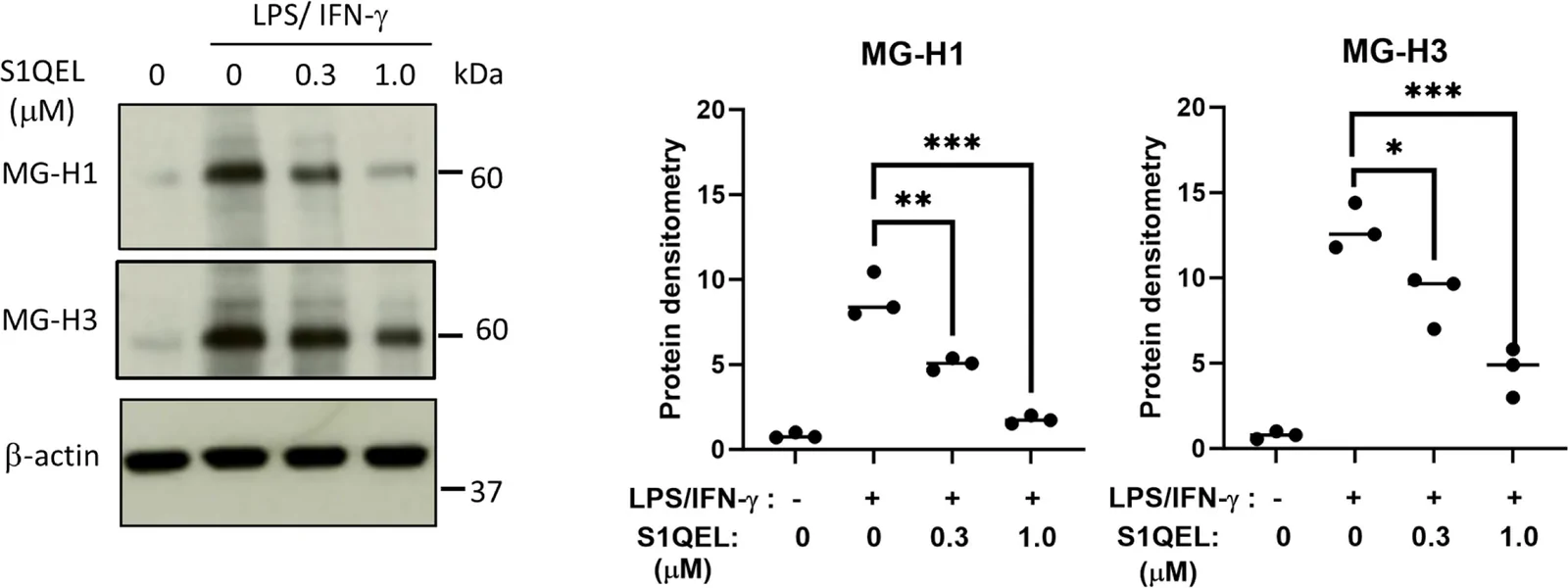
**Inhibition of mROS from complex I of the mitochondrial electron transport chain reduced accumulation of methylglyoxal.** WT mouse peritoneal macrophages were left untreated or stimulated simultaneously with LPS (10 ng/mL) and IFN-γ (20 ng/mL) for 24 hours, where indicated. Thirty minutes before this treatment, cells were treated with either vehicle control (DMSO) or the indicated doses of S1QEL1.1. Whole cell lysates were isolated at 24 hours poststimulation, and these lysates were subjected to Western blotting, probed with antibodies specific for MG-H1, MG-H3, and β-actin. The immunoblot images for all panels portray representative blots from three independent experiments. The graphs located to the right of the blots portray the mean densitometry values from each of these three experiments. * denotes *P* < 0.05. ** denotes *P* < 0.01. *** denotes *P* < 0.001. mROS, mitochondrial reactive oxygen species.

Finally, to determine whether S1QEL1.1 was sufficient to reduce the level of methylglyoxal, leading to accumulation of the cellular methylglyoxal-derived hydroimidazolones MG-H1 and MG-H3 in classically activated macrophages, whole cell lysates were analyzed. Targeting mROS in macrophages with S1QEL1.1 that were treated concurrently with LPS and IFN-γ significantly decreased the accumulation of both MG-H1 and MG-H3 (Figure 5).

## 4. Discussion

In recent years, host-targeted therapies have been proposed as novel treatments for patients experiencing sepsis ^[43]^. Given the continued paucity of effective treatments beyond conventional critical care interventions, targeting the hyperinflammation present in these trauma patients was seen as a potential novel alternative therapy. Despite success in preclinical trials using Eritoran to inhibit TLR4-dependent signaling in septic mouse models ^[44]^ and severe viral infections ^[45,46]^, Eritoran treatment did not achieve efficacy in a human phase III clinical trial for all-cause sepsis ^[47]^. Similarly, treatments antagonizing the activity of individual proinflammatory cytokines like interleukin (IL)-1β and tumor necrosis factor (TNF) α during sepsis were unsuccessful ^[48,49]^. In these cases, the intervention was potentially too limited in dose or timing. In the wake of such failures, an alternate hypothesis has been put forward that rewiring the metabolic pathways inside proinflammatory cells would allow them to repolarize into a less pathological, “tissue repair,” M2 phenotype, broadening the scope of the treatment. The metabolite methylglyoxal is formed during glycolysis and has been implicated as a biomarker for severe sepsis. Considering that glycolysis has been associated with enhanced inflammation, blunting the activity of HIF-1α, the master transcriptional regulator of glycolysis, would be predicted to be beneficial. Therefore, understanding how aerobic HIF-1α stabilization occurs and can be manipulated therapeutically represents a potential novel strategy to ameliorate the devastating effects of sepsis. Both S1QEL1.1 and S3QEL1.2 decreased the expression of IL-10 in peritoneal mouse macrophages induced by LPS treatment ^[50]^. Additionally, treatment with S3QEL1.2 caused downregulation of 146 genes, including IL-10, in bone marrow-derived macrophages treated with LPS for 24 hours ^[51]^. In astrocytes, S3QEL regulated 30% of response genes following IL-1α treatment, and it was suggested that the transcription factor STAT3 was mediating this effect ^[52]^.

Numerous lines of evidence have indicated that NO may play a role in this process. Ectopic overexpression of *Nos2* in a porcine kidney cell line (LLC-PK1) was sufficient to induce HIF-1α, and co-culturing nontransfected LLC-PK1 cells with the RAW 264.7 macrophage cell line stimulated with LPS and IFN-γ to induce NO formation led to HIF-1α stabilization in the LLC-PK1 cells that could be perturbed by addition of l-NAME ^[53]^. Doxorubicin-induced HIF-1α transcriptional activity was also decreased in the presence of l-NAME ^[54]^. Additionally, exogenous treatment of HEK293 cells with the NO donor *S*-nitrosoglutathione led to transcriptional activation of HIF-1α under normoxic conditions ^[55]^. Collectively, this data implies that NO is an important candidate for mediating the stabilization of HIF-1α. Previously, *N*-nitrosylation of a cysteine residue in HIF-1α was hypothesized to be directly required for this stabilization using a tumor radiotherapy model ^[56]^. Significantly, this novel mechanism was shown to be independent of PHD-dependent hydroxylation ^[56]^. Despite this evidence highlighting the contribution of NO to HIF-1α stabilization and activity, the regulation of HIF-1α by NO may vary depending on the oxygen tension. For example, iNOS was shown to repress HIF-1α transcriptional activity in C6 glioma cells during hypoxia ^[57]^.

Whether NO acts directly or indirectly in different inflammatory contexts remains unclear. Persistent exposure to NO can reversibly inhibit complex IV and forward electron flow of the mitochondrial electron transport chain ^[58]^. In macrophages, NO directly impairs the plasticity of this cell type by this respiration-blocking capacity, preventing repolarization to an anti-inflammatory M2 phenotype ^[37]^. This data suggests the possibility of an indirect mechanism whereby NO exerts its activity through the generation of mROS via inhibition of the electron transport chain, which is supported by previous studies that have shown that targeting mROS can alleviate HIF-1α stabilization ^[12]^.

**Figure 6.**
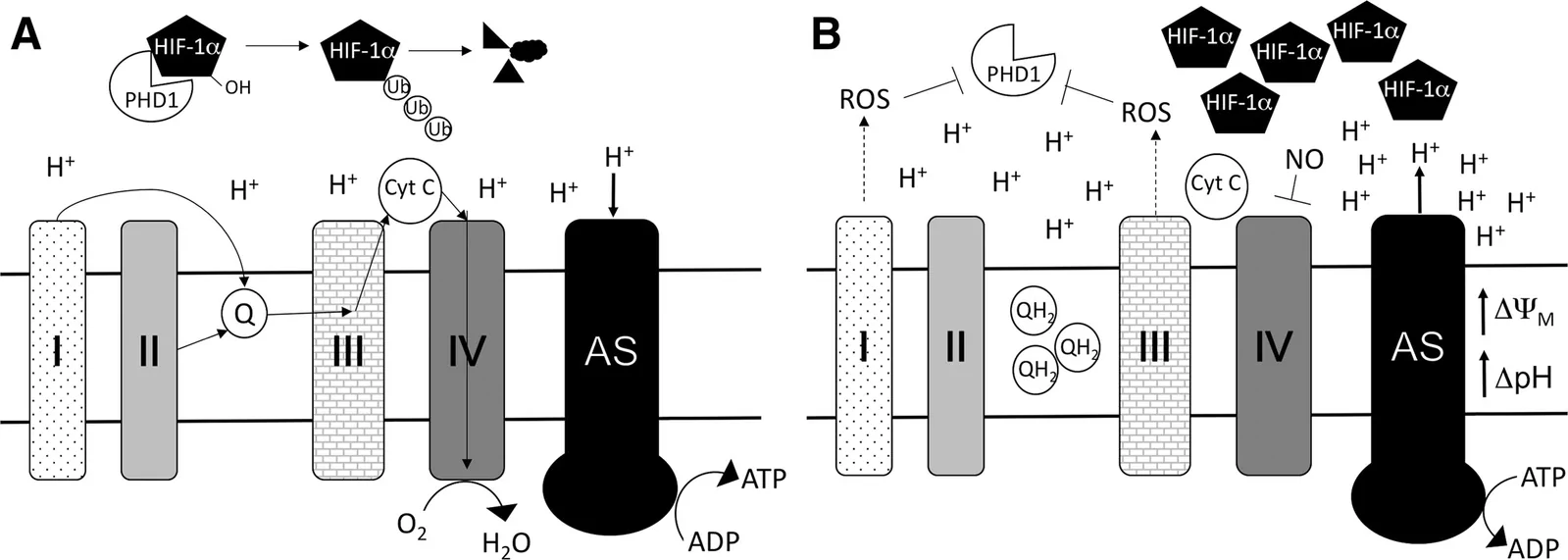
**Mitochondrial reactive oxygen species regulates stabilization of HIF-1**α **in classically activated macrophages: a model for our findings.** (A) In unstimulated macrophages, mitochondrial respiration occurs, a sequence of redox reactions involving four protein complexes (I-IV), ubiquinone (Q), and Cytochrome C (Cyt C) drive production of ATP through ATP synthase (AS) through the actions of a proton motive force. In the presence of oxygen, hydroxylation of HIF-1α by the PHD family of enzymes occurs, leading to HIF-1α ubiquitination and protein degradation. (B) In classically activated macrophages, nitric oxide (NO) accumulates, targeting complex IV in the electron transport chain, causing a back-up of electrons from upstream electron donors, facilitating accumulation of the reduced form of ubiquinone, ubiquinol (QH_2_). Additionally, ATP synthase- dependent hydrolysis of ATP derived from glycolysis generates a higher proton motive force due to the increased membrane potential (∆Ψ_m_) and pH gradient (∆pH). Together, the increased ubiquinol and high proton motive force cause complex I and complex III to overproduce mROS, which we hypothesize can inhibit PHD proteins by regulating the redox state of an essential iron ion that is required for its enzymatic activity. This ultimately leads to accumulation of HIF-1α and methylglyoxal even under normoxic conditions, which can be abrogated by blocking mROS with Mito-TEMPO, S1QEL, and S3QEL. ATP, adenosine triphosphate; HIF-1α, hypoxia-inducing factor-1α; mROS, mitochondrial reactive oxygen species; PHD, prolyl hydroxylases.

The concept of reverse electron transport was first discovered in 1961 and describes the flow of electrons counter to the direction used for ATP generation ^[59]^. In particular, reverse flux through complex I of the electron transport chain is associated with increased generation of mROS ^[60]^. One trigger of reverse flow through this complex is accumulation of reduced ubiquinone formed as a consequence of succinate oxidation at complex II of the electron transport chain ^[61]^. We speculate that NO inhibition of cytochrome oxidase prevents oxidation of the electron transport chain, leading to a greatly increased ubiquinol/ubiquinone ratio, and that high levels of glycolytically-derived ATP drive mitochondrial ATP hydrolysis to generate high proton motive force, which are the requirements for high ROS production by site I_Q_ of complex I and complex III ^[62–65]^. Blocking site I_Q_ with rotenone can impair IL-1β production in bone marrow-derived macrophages stimulated with LPS ^[12]^. ROS has been implicated in the regulation of PHD enzymes in neutrophils ^[66]^, suggesting a direct mechanism of action. However, this latter study also complicated matters by determining that ROS can arise through the glycerol-3 phosphate shuttle in the mitochondria ^[66]^. The limitation of approaches based on antioxidants such as Mito-TEMPO or MitoQ is that the targeting of the antioxidant to the mitochondria may not be specific and would likely not identify the exact source of ROS. Additionally, probes targeting mROS can yield dissimilar results as their signals are membrane potential-dependent and other ill-defined cellular redox reactions can alter the signal even if ROS/superoxide levels do not change. Work from the O’Neill lab has demonstrated that the addition of the nonspecific antioxidant *N*-acetylcysteine also limited HIF-1α activity, which was hypothesized to be exerted through regulation of succinate in classically activated macrophages ^[12]^. However, development of more specific tools would be required to properly explore the role of mROS on HIF-1α stabilization and build on this seminal finding. In this context, the suppressors of complex I and III mROS, S1QEL and S3QEL, allow the examination of mROS on a more granular level. Both S1QEL and S3QEL have been shown to decrease the expression of the anti-inflammatory cytokine IL-10 in peritoneal mouse macrophages induced by LPS treatment ^[50]^. Additionally, treatment with S3QEL1.2 caused downregulation of 146 genes, including IL-10, in bone marrow-derived macrophages treated with LPS for 24 hours ^[51]^. In astrocytes, S3QEL regulated 30% of response genes following IL-1α treatment, which was suggested to be mediated in part through regulation of the transcription factor signal transducer and activator of transcription (STAT) 3 ^[52]^. Finally, S3QEL impairs HIF-1α stabilization under hypoxic conditions ^[30,31]^. However, the effect of these chemicals on HIF-1α stabilization in macrophages under aerobic conditions was not characterized. Our data indicate that NO-induced mROS originating from complex I and complex III is the source of ROS that can regulate HIF-1α, and we hypothesize that this effect is exerted through antagonism of PHD enzymes by reduction of an essential iron ion in these proteins (Figure 6). Methylglyoxal can perturb mitochondrial function following glycation of complex III proteins and generate superoxide in a rat model of diabetes ^[67]^. This suggests that methylglyoxal may further regulate mROS in a feed-forward manner after NO has exerted its effect on HIF-1α.

Repurposing of mitochondrial ATP synthase from ATP generation to ATP consumption may have significant roles in macrophage biology. Importantly, the generation of mROS through this process has been shown to contribute to activation of the NLRP3 inflammasome and the ensuing release of IL-1β ^[68]^. Although mROS generated downstream of TLR4-dependent signaling was shown to help facilitate clearance of *Salmonella typhimurium* ^[69]^, this process is not exclusively beneficial. In addition to this effect on host signaling pathways, mROS can also play a pathogenic role during *Mycobacterium tuberculosis* infection, causing necrosis of infected macrophages ^[70]^. Excess mROS may also help sustain chronic inflammation in myeloid cells that underlies various pathologies. For example, blocking mROS generated at complex I in human and mouse cells in vitro limited neurotoxicity ^[71]^. Additionally, mice carrying a mutation in the complex I gene *Nd6* that functionally blocks mROS formation ^[72]^ exhibited a milder course of Experimental Autoimmune Encephalomyelitis than control mice ^[71]^. Blocking mROS may also underlie the anti-inflammatory effects of exogenous itaconate in vitro and in vivo ^[23]^. Overall, these data raise the possibility that targeting this axis during sepsis may prove beneficial and that methylglyoxal produced by myeloid cells may serve as a biomarker for cells chronically sustaining deleterious inflammation.

Our gene expression data indicate that ROS generated from complexes I and III regulate a subset of genes in classically activated macrophages, allowing S1QEL and S3QEL to potentially serve as therapeutics in cases of sepsis through suppression of this ROS. Theoretically, this should broaden the desired scope of treatment to treat sepsis compared with therapies targeting individual cytokines like TNF-α or IL-1β. However, despite its link to deleterious inflammation during sepsis, HIF-1α has also been implicated in the expression of costimulatory molecules in dendritic cells stimulated with LPS ^[73]^. Similarly, HIF-1α is important for mediating IFN-γ-dependent host defense during infection with *Mycobacterium tuberculosis* ^[74]^, highlighting the caution needed when considering whether this is an effective target during an immune response. Further studies will be needed to characterize whether rewiring metabolism using these compounds during sepsis is safe and effective.

Finally, our study does present specific limitations that should be considered when analyzing its impact. The absence of direct mROS measurements following treatment with mROS antagonists is one prominent example. Additionally, the determination of HIF-1α stabilization cannot be conclusively proven without further analysis of prolyl hydroxylation and the binding of cellular degradation machinery and may instead represent increased total protein expression levels of HIF-1α.

## 5. Conclusions

Our data support a model in which NO-mediated mitochondrial dysfunction shapes macrophage inflammatory responses by increasing mROS, promoting HIF-1α stabilization and methylglyoxal accumulation. Although targeting mROS may offer a therapeutic strategy to improve sepsis outcomes, further studies will be needed to characterize whether rewiring metabolism using these compounds during sepsis is safe and effective.

## Author contributions

DP and SNV designed the research and wrote the paper. DP and JCP conducted the research. MDB and MAW provided essential reagents. DP analyzed data and performed statistical analysis and primary responsibility for final content. All authors read and approved the final manuscript.

## Conflicts of interest

The authors declare that they have no conflicts of interest.

## Funding

This study was supported by grant support from the NIH AI174374 (DP and SNV). The content is solely the responsibility of the authors and does not necessarily represent the official views of the National Institute of Health. Additionally, the National Institute of Health was not involved in the preparation or made restrictions regarding publication of the manuscript.
